# Micro-Ring Resonator Assisted Photothermal Spectroscopy of Water Vapor

**DOI:** 10.3390/s24113679

**Published:** 2024-06-06

**Authors:** Maria V. Kotlyar, Jenitta Johnson Mapranathukaran, Gabriele Biagi, Anton Walsh, Bernhard Lendl, Liam O’Faolain

**Affiliations:** 1 Centre for Advanced Photonics and Process Analysis, Munster Technological University, Bishopstown, T12 P928 Cork, Ireland; jenitta.johnsonmapranathukaran@mycit.ie (J.J.M.); gabriele.biagi@mtu.ie (G.B.); anton.walsh@mtu.ie (A.W.); william.whelan-curtin@mtu.ie (L.O.); 2 Tyndall National Institute, Dyke Parade, T12 PX46 Cork, Ireland; 3 PolySense Lab, Dipartimento Interateneo di Fisica, University and Politecnico of Bari, Via Amendola 173, 70126 Bari, Italy; 4 Institute of Chemical Technologies and Analytics, TU Wien, Getreidemarkt 9/164, 1060 Vienna, Austria; bernhard.lendl@tuwien.ac.at

**Keywords:** integrated photonics, photothermal spectroscopy, refractive index sensing

## Abstract

We demonstrated, for the first time, micro-ring resonator assisted photothermal spectroscopy measurement of a gas phase sample. The experiment used a telecoms wavelength probe laser that was coupled to a silicon nitride photonic integrated circuit using a fibre array. We excited the photothermal effect in the water vapor above the micro-ring using a 1395 nm diode laser. We measured the 1f and 2f wavelength modulation response versus excitation laser wavelength and verified the power scaling behaviour of the signal.

## 1. Introduction

Spectroscopic analysis of gaseous species is very important in the determination of the chemical composition of a gas sample. Different molecules and atoms absorb and emit light at specific wavelengths, allowing the identification of substances present in the gas, along with their concentration, by studying the characteristic absorption spectrum. Spectroscopic analysis of gases allows monitoring of environmental changes [1] and pollution levels, including the identification of pollutants, monitoring greenhouse gas concentrations [2], and assessing air quality [3]. In industry, spectroscopic analysis for process control is important for ensuring the quality of materials and for monitoring chemical reactions, including the gases in combustion processes [4], the plasma used in manufacturing processes [5], and studying microbiological processes [6].

The absorption of electromagnetic energy by molecules excites their internal energy levels, which leads to sample heating as the molecules relaxe non-radiatively. This photo-induced temperature change causes, in turn, changes in the sample’s density and pressure, which can be used for the spectroscopic analysis of the sample. For example, photoacoustic spectroscopy monitors the generated acoustic wave [7] and photothermal spectroscopy (PTS) monitors the refractive index wave [8]. In particular, the refractive index of a substance is a volume-independent quantity, which means the measured signal is not necessarily affected by sample volume, unlike intensity-based measurements, making the photothermal effect very promising for the realization of miniaturized sensors and/or sensors that can work with extremely small sample volumes.

The refractive index transducer that converts the photothermally induced effects into a measurable signal is a key element of a PTS system. The induced changes in the refractive index are tiny (on the order of 10^−11^ to 10^−12^ refractive index units) and thus the transducer must be extremely sensitive. Photothermal mirrors [9] and photothermal lensing [10] have been used to measure the photothermal change in angle of a reflected or refracted beam. Lendl et al. have used a high finesse Fabry–Perot etalon (with typically a finesse of ~100 and cavity length of 1 mm) to implement the interferometric cavity-assisted PTS for a wide range of gas samples [11]. The photothermally induced change in refractive index shifts the periodic transmission function of the etalon, which is then monitored via a change in the transmitted intensity of the probe. Recently, Krzempek et al. have used miniaturized fibre-based Fabry Perot cavity sensors to retrieve the PTS signal in a proof-of-concept measurement of methane [12]. Similarly, Njegovec et al. have demonstrated an all-fibre PTS gas sensor, comprising a probe fibre, a slotted capillary and an excitation fibre [13], and Yao et. al. used a fibre-based PTS [14] with a hollow core anti-resonator mid IR fibre.

Micro-ring resonators [15] can provide similar, or better, finesse to the etalons used above and have been used for PTS in solid-phase polymers [16,17]. Silicon photonics offers considerable potential for miniaturisation and mass production, with a range of components including spot size converters [18], fibre pig tailing [19], high quality-factor (Q-factor) micro-ring resonators [20] and on-chip lasers [21], but its full potential for on-chip PTS is yet to be realised. Recently, Yan et al. have used a ~90 mm long lithium niobate rib waveguide to detect CO_2_ with an 870 ppm detection limit via the photothermal effect, demonstrating the potential of this line of research [22].

In this work, we demonstrated micro-ring resonator (MRR) assisted photothermal spectroscopy for gas phase samples for the first time. We used a fibre coupled silicon nitride micro-ring resonator as the refractive index transducer with a telecommunications wavelength probe laser and a 1395 nm diode laser to excite and measure the photothermal effect induced in water vapour for a range of excitation laser powers. Quantitative analysis of water vapour is a frequently encountered analytical task that is very relevant in many different applications scenarios such as industrial process monitoring [23,24], quality control of medical gases as well as in environmental monitoring and research [25].

Despite the almost ubiquitous need for highly selective moisture sensors [26], current sensor technologies are mainly based on the detection of a changing property of a sensor element with limited selectivity towards the target analyte water. Examples are capacitive sensors based on ceramics or polymers, resistive sensors employing semiconductors as well as chilled mirror dew point sensors. The advantages of these established solutions for detecting water vapor are their small size and often ease of integration into a given industrial apparatus or pipe system. Encountered problems are, however, cross sensitivity towards other volatile and polar substances such as glycol, methanol or amines, limited dynamic range and drifts. For highly selective trace analysis of water vapor, tuneable diode laser absorption spectrometers are also in use [27]; however, due to their size they can hardly be considered sensors that can be used in in-line sensing applications. In this context, we envision that integrated PTS based gas sensor technology might be developed into a powerful solution as it promises inherently high selectivity with a small footprint for a possible seamless integration and use in many different application scenarios. This manuscript reports on our first efforts towards reaching this goal.

## 2. Sensing Principle

**Figure 1:**
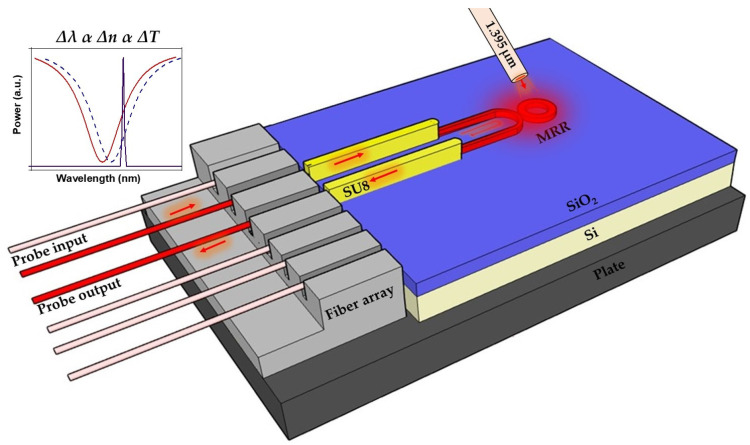
Schematic of the experimental set-up showing the light from the telecom wavelength probe laser delivered via input fibre through the sport size converter (SU-8) to the micro-ring resonator (MRR) and out again through the output fibre; the excitation beam at 1395 nm is delivered via the out-of-plane fibre, which is placed over the MRR. The insert shows the sensing principle where the shift in the resonance of the MRR (original in red, shifted curve in blue) is caused by the change in the refractive index of the air above the ring, which is caused in its turn by the change in the temperature. The probe laser at the telecom wavelength (black) is matched to the inflection point of the resonance.

Figure 1 shows a schematic of the measurement principle. The excitation beam excites the analyte above the ring resonator by means of a lensed optical fibre. The near-infrared probe beam (in the telecommunications C-band) is coupled to the silicon-based chip containing the silicon nitride (SiN) photonic integrated circuit via a fibre array (FA), with the SiN waveguides looped back appropriately. The use of a fibre array is an important technological advance as optical access is thus required only to one facet of the chip, simplifying the configuration of the measurement setup. The resonance ($\lambda_{res})$ of the micro-ring has a Lorentzian line shape characterized by the q-factor, extinction ratio and centre wavelength given by (1):

(1) $$m\lambda_{res}=2\pi Rn_{eff}$$

where *m* is the mode number, *R* the ring waveguide radius and $n_{eff}$ the effective refractive index. The absorption of the excitation beam by the analyte creates a temperature change that induces a change in the effective refractive index. The wavelength of the probe is set to the inflection point of the Lorentzian resonance, and the modulation of the excitation beam creates a modulation of the probe that can be detected using a photodiode and lock-in detection scheme. By scanning the wavelength of the excitation beam, the absorption spectrum of the analyte can be recovered and thus the analyte identified, and its concentration quantified.

The measured signal is proportional to the photoinduced temperature change ΔT, which is in turn proportional to the excitation laser power, the optical absorption coefficient, the sample concentration and scales inversely with the square of the excitation beam radius, the sample density and heat capacity [28]. Full modelling of the photothermal effect is beyond the scope of this paper, as the effects of the silicon chip complicate the analysis, but a discussion of its effects on excitation beam diameter and on the generated photothermal signal can be found in [15].

## 3. Fabrication

**Figure 2:**
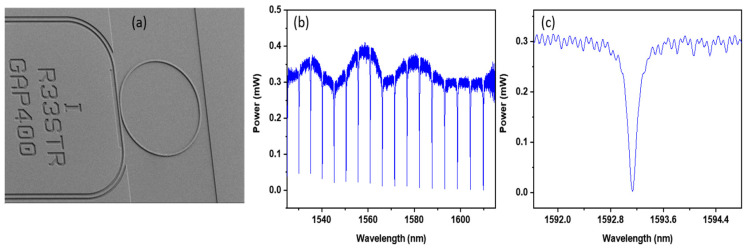
(**a**) A SEM image of the fabricated MRR with looped access waveguides, (**b**) The transmission spectra of the MRR, (**c**) The selected resonance of the MRR with a quality factor Q of 20,000.

A bulk 4” silicon wafer was thermally oxidized to produce a 2.2 µm SiO_2_ layer on the top of the wafer. A SiN layer of 300 nm thickness was deposited by plasma enhanced chemical vapor deposition (PECVD), which was subsequently spin coated with a layer of 450 nm thick ZEP 520A electron-beam resist. An Elionix electron-beam lithography system operating at 100 kV was used to define the MRR pattern. After development in a bath of ZED solution, the pattern was transferred to the SiN layer by inductively coupled plasma (ICP) etching with CHF_3_:O_2_ chemistry at an etch rate of 1.25 nm/s. The remaining resist was washed away in a bath of 1165 solvent at 90 °C. SU-8 resist of 3 µm thickness was spun on the top of the sample with the fabricated MRRs. A second stage electron beam lithography process was performed in order to define 3 μm wide SU-8 waveguides positioned over the silicon nitride access waveguides. The SiN waveguides, which were exposed during first stage exposure, were adiabatically tapered from 200 nm to 1.1 μm width over the length of 500 µm to create inverse taper spot-size converters [29] to implement efficient coupling between the fibre array and the MRRs. The transmission spectra (Figure 2b,c) of the fabricated MRRs (Figure 2a) with spot size converters were obtained using an Optical Spectrum Analyzer (OSA, Yenista optics, OSA20).

## 4. Measurement Setup

**Figure 3:**
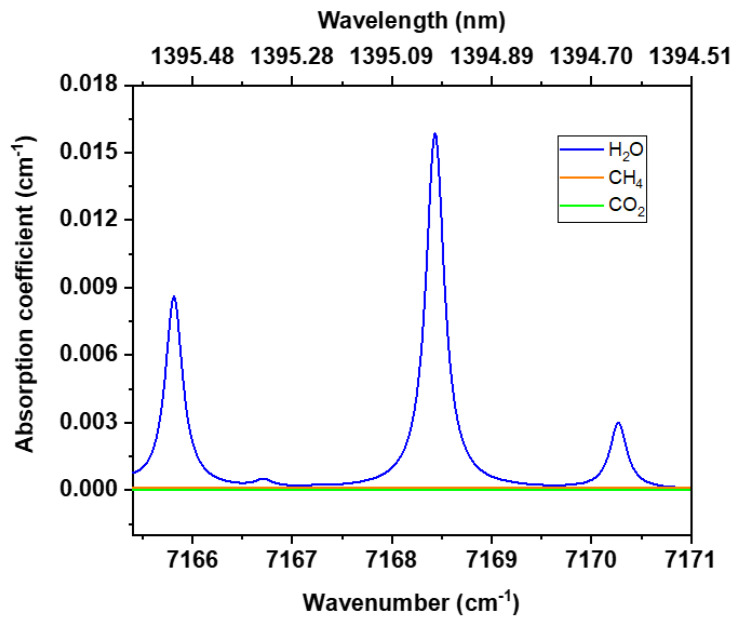
Near-IR absorption spectrum of air at normal pressure. The absorption of the main IR active gases present at their natural abundances are shown. Over this wavenumber range, the absorption of methane and carbon dioxide at atmospheric concentrations is very small compared to water (~5 orders and 7 orders of magnitude less, respectively). The data was taken from the HITRAN database [30].

A fibre pigtailed laser diode (Eblana Photonics, EP1395-0_DM_B01_FADES4082, Dublin, Ireland) of wavelength λ = 1395 nm was used as the excitation source. The laser targets the water (H_2_O) absorption line of the fundamental band falling at 7168.43 cm^−1^ (Figure 3). The absorption cross section of the transition was retrieved from the HITRAN database [30] and has a maximum value of 1.5 × 10^−22^ cm^2^/molecule. The simulated absorption spectrum given in Figure 3 shows the absorption spectra of the main IR active gases present in the atmosphere (water, carbon dioxide and methane) according to their natural abundances and assuming a line broadening typical for air at normal pressure. From this data, it is evident that the chosen transition of the water molecule at 7168.433 cm^−1^ is not obscured by other IR active molecules in air. Therefore, by utilizing the narrow emission of the selected laser diode it is possible to probe the water vapour in air in a highly selective manner.

A fibre array (8 channels, SMF-28) with a pitch of 127 µm was used to couple the telecoms wavelength probe laser to the chip. SU-8 spot size converters were used to adapt the mode to that of the SiN waveguide with a fibre-to-fibre loss of 9.1 dB.

**Figure 4:**
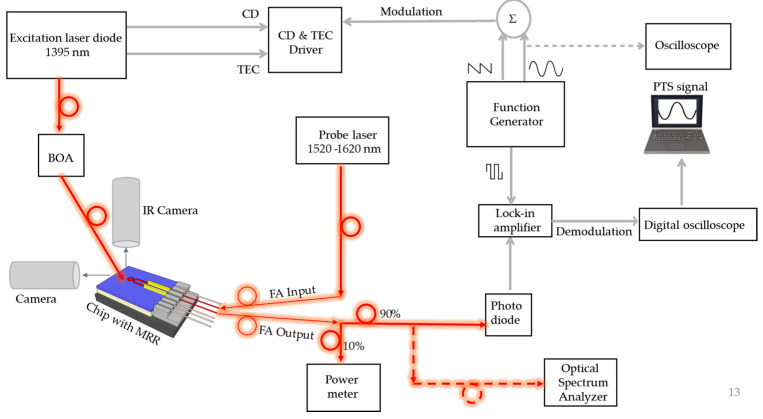
A schematic of the experimental setup for the investigated gas sensor system (BOA = booster optical amplifier, CD = current driver, TEC = thermo-electric cooler, IR = infra-red, FA = fibre array, MRR = micro-ring resonator).

The MRRs used in the experiments had a radius of 33 μm and quality factor of ~20,000. The light from the excitation laser was directed via a lensed fibre attached by a custom-made 3D printed tube to a high-precision Thorlabs rotating stage (PR01), allowing the use of different angles between the fibre and the surface of the MRR. The position and focus of the excitation laser beam were optimized with the help of an Infra-Red (IR) camera (Photonic science SWIR3151, Hastings, UK) from the top and the height of the fibre with respect to the sample surface was estimated using a visible camera (Thorlabs, DCC1645C, Newton, NJ, USA) from the side (Figure 4). A Laser Diode Controller (LDC, Stanford research systems, LDC501, Sunnyvale, CA, USA) with an inbuilt Thermo-Electric cooler (TEC) was connected to the excitation laser using a butterfly mount (Thorlabs LM14S2) to stabilize the temperature and drive the injection current. The laser diode has a threshold current of 15 mA at 26 °C and a slope efficiency of 0.15 mW/mA. The maximum H_2_O absorption was observed at an injection current of 102.4 mA of the excitation laser with an emitted optical power of 10 mW measured by the power meter (PM, Thorlabs, PM100D).

A booster optical amplifier (BOA, Thorlabs BOA1410P) was driven by a separate TEC and LD controller (Thorlabs CLD1015) and used to vary the output power of the excitation laser. A sinusoidal signal of frequency 6 kHz was applied as the external amplitude modulation to the excitation laser through the LDC. The digital version of the same signal was then given as the trigger to the Lock-In Amplifier (LIA, Stanford research systems, SR830 DS). A slow voltage ramp (sawtooth signal) of periods between 60 and 180 s was then applied to scan the laser current in time. Both signals were generated from a two-channel Function Generator (FG, Lamda, Rigol DG812, Harpenden, UK) and observed in an oscilloscope (Rigol DS1102E). Simultaneously, the output from a broadband (1520–1630 nm) tuneable laser source (TLS, Yenista Optics Tunics- T1005-HP, Princeton, NJ, USA) was launched as a probe signal via a fibre array to and from the MRR. Its output was collected by a InGaAs-based amplified photodetector (PD, Thorlabs PD10CS2) via a 90:10 fibre optic splitter where 90% of the power was connected as the signal input to the LIA and the other 10% was connected to the power meter to observe the resonance of the MRR. The signal, which was sent to the LIA, was demodulated at the 1st and 2nd harmonic. The integration time varied between 300 ms and 1 s. The output from the LIA was converted using a Digital Oscilloscope (Pico technology, Picoscope R4262, St Neots, UK) and then recorded by Picoscope 6 software in a laboratory computer.

The fibre array, the MRR chip and the rotating stage for the fibre carrying the excitation laser light were mounted on three separate three-axis translation stages (Thorlabs, MBT616D) to guarantee an accurate alignment between the sample and the probe/excitation lasers beams. The gap between the excitation fibre and the chip was approximately 10 microns.

## 5. Results

The generation and identification of PTS signals involves transient heating of the sample gas. Wavelength modulation (WM) spectroscopy, wherein a tuneable laser’s emission frequency undergoes periodic modulation, is a particularly attractive means of implementing the required periodic heating, as it can significantly increase the signal to noise ratio [31]. The transducer signal is then detected by a lock-in amplifier at typically the first or the second harmonic (1f, 2f) of the modulation frequency. This technique is widely used in trace gas detection, for example, in laser absorption spectroscopy [32] and quartz enhanced photo-acoustic spectroscopy [33,34] and interferometric cavity-assisted photothermal spectroscopy [11,35]. The detection of second harmonic components produces a signal proportional to the second derivative of the absorption spectrum. The 2f-WM scheme also eliminates linear slopes in the spectra, which suppresses background absorption from the broad featureless bands as only non-linear absorption features generate a signal.

**Figure 5:**
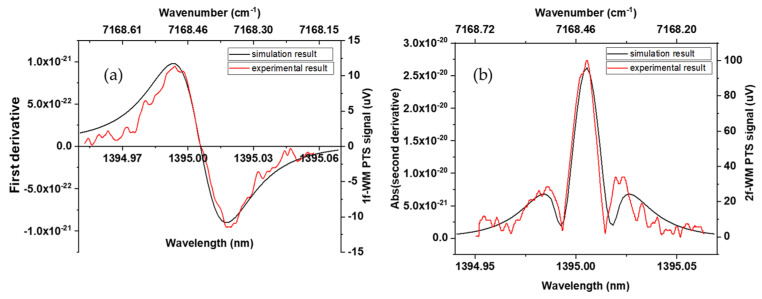
Spectra of 1f-WM PTS (**a**) and 2f-WM PTS (**b**) signals recorded as a function of the excitation laser wavelength. Black lines are the theoretically predicted 1f and 2f spectra.

To make the first-ever experimental results on the PTS of gas samples using an on-chip MRR as the transducer, we recorded both the first and second harmonics of the PTS signal using a lock-in amplifier, Figure 5. As one can see from these graphs, the maximum of the 2f PTS signal and the zero position of the 1f PTS signal was achieved at the excitation laser driver current of 102.4 mA, which corresponds to the maximum of the water vapor absorption at a wavelength of 1395 nm (Figure 3). A sinusoidal dither was used to perform wavelength modulation. By applying a sinusoidal waveform (6 kHz and 120 mV peak-to-peak) to the laser driver, the emission wavelength of the laser is swept over a small range around the wavelength of the absorption peak (+/−0.026 nm). The full absorption feature is scanned by applying a 10 mA ramp with a period of 150 s to the laser.

The probe laser wavelength was kept at the inflection point of one of the resonance peaks of the MRR. The influence of the probe laser wavelength on the strength of the PTS signal will be discussed later in this section.

The measured spectra in Figure 5 show characteristic shapes that are in good agreement with those reported in the literature [36]. The first and second derivatives of the absorption spectrum of water, to which the 1f-WM and 2f-WM intensities are proportional, are shown in black in Figure 5a,b for reference.

To improve the signal-to-noise ratio and investigate the influence of the excitation laser power on the PTS signal, we performed optical amplification of the excitation laser by means of the booster optical amplifier. The output power of the excitation beam, after the BOA, was thus varied from 11 mW at 80 mA of current to 138 mW at 440 mA of current.

**Figure 6:**
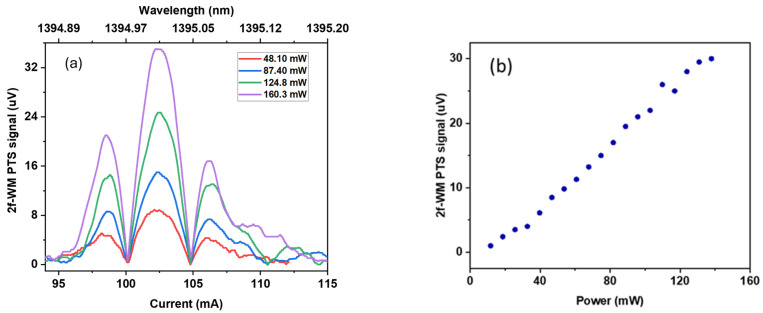
(**a**) 2f-WM PTS signal at different powers of the amplified excitation source. (**b**) Maximum 2f-WM PTS signal as a function of excitation power.

In Figure 6a, the 2f-WM PTS signal is shown for a selection of output powers of the excitation laser taken over 60 s of a long current sweep. Figure 6b shows a plot of the maximum PTS signal at different excitation powers, i.e., the excitation laser wavelength was kept constant at the centre of the targeted gas absorption line and the probe wavelength was kept at the MRR inflection point for both parts of this experiment. The near-linear dependence of the amplitude of the 2f-WM PTS signal on the excitation power can be seen in Figure 6b, which is consistent with the equation (2) for the amplitude of the photo-induced signal (*S*) [28], where *ε* is the molar absorption coefficient and *c* the concentration of the analyte gas, and *P* and *f* are the optical power and modulation frequency correspondingly of the excitation light, and *V* is the interaction volume:

(2) $$S\sim \frac{\varepsilon \cdot c\cdot P}{V\cdot f}$$

The demonstrated linear dependence of the signal on the excitation laser power in Figure 6b serves as proof that we measured the water vapor by means of our PTS sensor.

**Figure 7:**
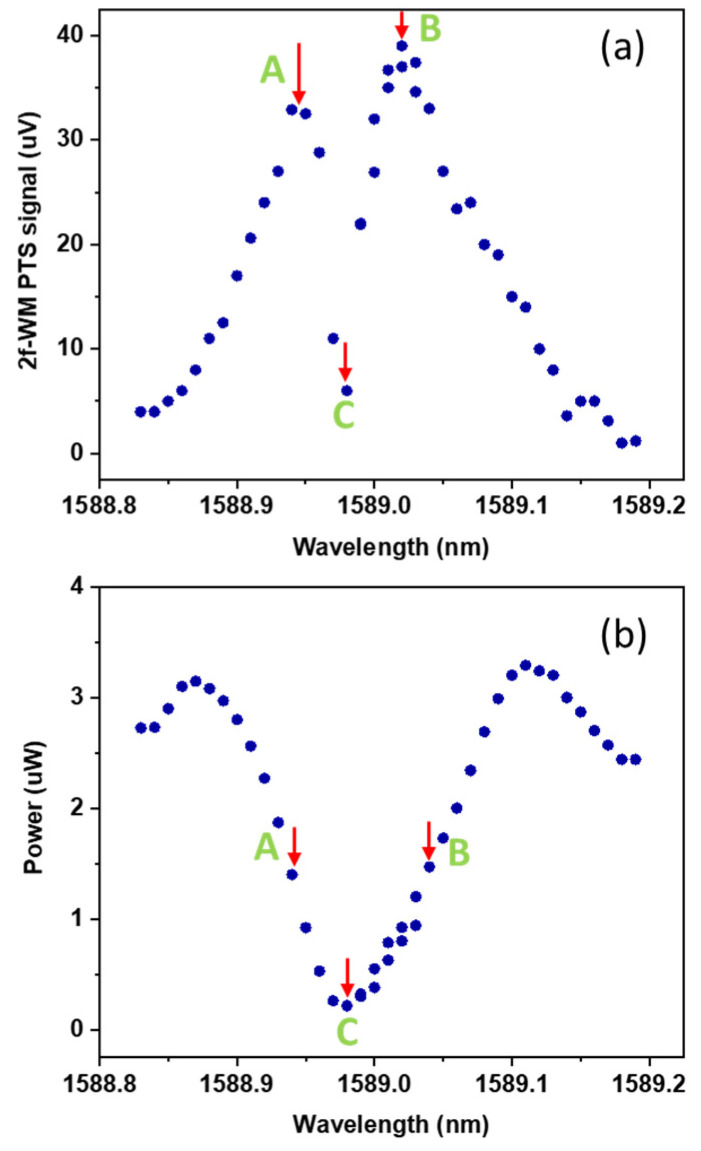
(**a**) 2f-WM PTS signal as a function of the probe wavelength where points marked as A and B correspond to the inflection points of the resonance dip and the point C is at the resonance wavelength. (**b**) The resonance dip of the MRR along which the 2f-WM PTS signal from part (**a**) was scanned.

In Figure 7, we investigated the influence of the wavelength of the probe laser with respect to the resonance spectrum of a chosen peak while keeping the excitation wavelength constant at the water vapor absorption maximum at 1395 nm. The BOA current was set at 400 mA, producing a 124 mW excitation beam. Figure 7b shows one fully resolved resonance peak of the MRR and the probe wavelength was scanned along the shape of the resonance. The resulting 2f PTS signal is depicted in Figure 7a, where the maximum PTS signal is observed at two inflection points on either side of the resonance peak and the minimum of the 2f PTS signal was recorded at the resonance. A decay in the amplitude of the 2f PTS signal is observed as the probe wavelength moves beyond the inflection points and away from the peak of the resonance where the MRR shows a flat response. This is the expected behaviour of the PTS signal as setting the probe laser to the inflection point produces the strongest PTS signal as it shows the largest rate of change in the measured probe laser intensity and the photothermal response undergoes the most significant shift. Consequently, taking measurements close to this point provides high sensitivity of photo-thermal detection. We did not observe any thermal effects arising from the absorption of the probe laser light by the micro-ring. Silicon nitride is essentially transparent at the probe wavelength and exhibits very low two photon absorption (unlike silicon, for example). We made measurements with probe powers in the 0.2 to 20 mW range and observed no changes to the profile of the recorded 2f spectrum.

In case of weak heating of the whole chip due to the minimal residual absorption of the excitation laser by the silicon substrate chip, we would expect a flat, uniform response as the residual absorption can be assumed to be constant in the wavelength range covered during the photothermal experiment, as when performing current tuning of the excitation laser a spectral region less than 1 nm is covered. We noted the 2f WM eliminates any background that may arise from broadband absorption. We observed signals at the 1st and 2nd harmonic of the applied modulation frequency, which we attributed to the periodic crossing of the laser line over the narrow resonant transition of the water vapor. This periodic process generates periodic heating, which is detected and amplified by lock-in detection. Therefore, we may conclude that residual heating, which might be present to a small extent, does not adversely affect the detection of water vapor above the ring resonator.

One should note that during the measurements, the peak of the resonance exhibited a slow drift due to local temperature fluctuations, resulting in a slight asymmetry in the curve.

## 6. Discussion and Conclusions

In this work, we report a photothermal spectroscopy method that uses a micro-ring resonator as the refractive index transducer. We demonstrated the measurement of a gas phase sample for the first time with such a system. As a proof-of-principle, we measured the 1f and 2f WM spectra of water vapor at atmospheric pressure. We verified the predicted power scaling dependence of the signal by varying the excitation beam power by means of a booster optical amplifier. The system is compact, based on diode lasers, and has excellent potential for further miniaturization. The use of a fibre array is an important technological advance as optical access is required only from one facet, which creates greater flexibility in the experimental configuration. The design can be fabricated using standard silicon fabrication techniques, giving a clear route to mass manufacture [37]. Recent advantages in photonic packaging [38] allow the realization of robust packages by means of fibre array attachment using epoxy bonding [39].

The stability and sensitivity can be improved through the implementation of a control loop to lock the probe laser to the inflection point of the MRR resonance and through the creation of a suitable enclosure to reduce the effects of environmental noise on the setup. Similarly, by mounting the excitation fibre in a more stable manner the effects of external acoustic noise will be reduced. Operating at reduced pressures will increase the 2f-WM signal by reducing the linewidth of the absorption curve and thus increase its steepness. Sensitivity can also be improved by using resonators with higher Q-factors. Furthermore, as the photothermal signal is proportional to the light intensity at the point of excitation, the use of more compact refractive index sensors [40] will allow further reductions in the excitation volume and will improve the intensity, giving significant improvements in the sensitivity.

## Data Availability

Data available within the article. Raw data will be made available upon request.
